# Evaluation of the diagnostic performance of a decision tree model in suspected acute appendicitis with equivocal preoperative computed tomography findings compared with Alvarado, Eskelinen, and adult appendicitis scores: A STARD compliant article: Erratum

**DOI:** 10.1097/MD.0000000000018733

**Published:** 2019-12-27

In the article, “Evaluation of the diagnostic performance of a decision tree model in suspected acute appendicitis with equivocal preoperative computed tomography findings compared with Alvarado, Eskelinen, and adult appendicitis scores: A STARD compliant article”,^[1]^ which appeared in Volume 98, Issue 40 of *Medicine*, affiliation e is incorrectly listed and should appear as “Department of Emergency Medicine, Dankook University School of Medicine, Kangwon National University, South Korea.”
